# Diagnostic Value of Salivary Markers in Neuropsychiatric Disorders

**DOI:** 10.1155/2019/4360612

**Published:** 2019-05-02

**Authors:** Agnieszka Kułak-Bejda, Napoleon Waszkiewicz, Grzegorz Bejda, Anna Zalewska, Mateusz Maciejczyk

**Affiliations:** ^1^Department of Psychiatry, Medical University of Bialystok, 16-070 Choroszcz, Poland; ^2^Department of Human Philosophy and Psychology, 15-295 Białystok, Poland; ^3^Department of Restorative Dentistry, Medical University of Bialystok, 15-276 Bialystok, Poland; ^4^Department of Physiology, Medical University of Bialystok, 15-222 Bialystok, Poland

## Abstract

A growing interest in the usability of saliva has been observed recently. Using saliva as a diagnostic material is possible because it contains a varied range of composites, organic and inorganic like proteins, carbohydrates, and lipids, which are secreted into saliva. Moreover, this applies to drugs and their metabolites. Saliva collection is noninvasive, and self-collection is possible. There is a lack of risk of injuries related to injection with needle, and it is generally safe. Human saliva has been successfully used, for example, in the diagnosis of many systemic diseases like cancers, autoimmunological diseases, infectious diseases (HIV, hepatitis, and malaria), and endocrinological diseases, as well as diseases of the gastrointestinal tract. Also, it is used in toxicological diagnostics, drug monitoring, and forensic medicine. The usefulness of saliva as a biological marker has also been extended to psychiatry. The specificity of mental illness and patients limits or prevents cooperation and diagnosis. In many cases, the use of saliva as a marker seems to be the most sensible choice.

## 1. Introduction

At present, growing interest in the usability of saliva has been observed [1–4]. Human saliva takes part in the protection against different pathogens of oral tissues and upper respiratory and digestive systems [1, 2].

One of the most important roles of saliva is to provide the right environment for oral mucosa and teeth. It protects against the variability of destructive biological or chemical substances and mechanical damage. Also, saliva plays a significant part in the primary phase of digestion and participates in the perception of different kinds of tastes. Moreover, saliva has antibacterial, antifungal, and antiviral properties due to the presence of immunoglobulins, lactoferrin, and lysozyme [4–6].

Using saliva as a diagnostic material is possible because it contains a varied range of composites, organic and inorganic like proteins, carbohydrates, and lipids, which are secreted into saliva. This also applies to drugs and their metabolites [6–10]. Its components are very sensitive, and they have a great response to toxic substances. They also correlate to the real-time level of these markers. Moreover, saliva collection is noninvasive, and self-collection is possible. There are no risk of injuries related to injection with needle, and it is generally safe [2, 11, 12].

**Figure 1:**
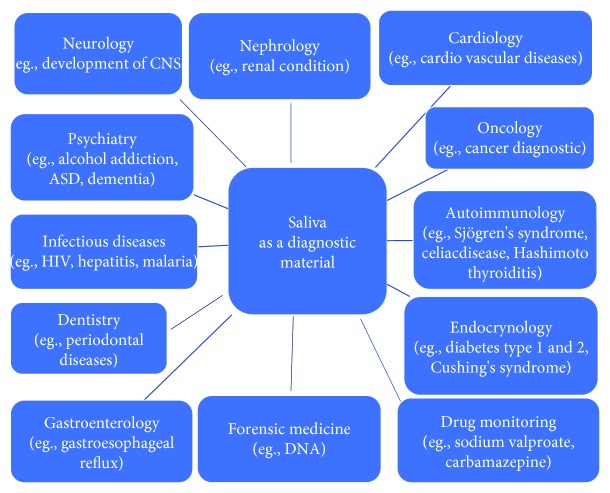
Saliva as a diagnostic material in medicine.

Hence, many studies recommended saliva as the model of noninvasive diagnostic material. Nowadays, human saliva might be used in the monitoring and the early diagnosis of different systemic diseases, such as infectious cardiovascular disorders and cancers [6, 13]. Analysis of the concentrations of various salivary components is becoming increasingly important in laboratory medicine and the monitoring of the therapeutic range of drugs [6, 14–19]. Currently, saliva is used in toxicological diagnostics, e.g., detection of drug dependence and alcohol abuse [2, 5, 6, 11, 20–22], neurology, psychiatry [6, 23–25], and forensic medicine (DNA) [26] (Figure 1).

**Figure 2:**
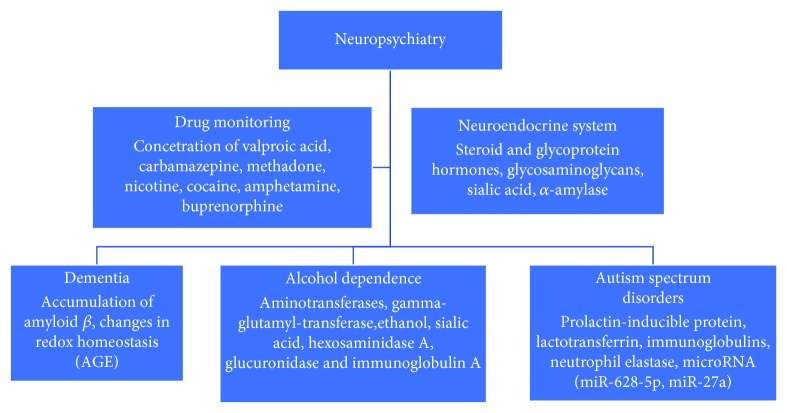
Saliva as a diagnostic material in neuropsychiatry.

In recent years, the usefulness of saliva as a biological marker has also been extended to psychiatry. The specificity of mental illness and patients limits or prevents cooperation and diagnosis. In many cases, the use of saliva as a marker seems to be the most sensible choice (Figure 2).

## 2. Drug Monitoring

It was proved that the concentrations of drugs in saliva correlate with the level of the drug in the blood [6, 27–31]. Therapeutic drug monitoring is used to optimize the management of patients receiving drug therapy. It encompasses the quantity of drug concentrations in biologic fluids. It also correlates with the patient's clinical condition and helps recognize the need to change the dosage, for example. Saliva use in drug monitoring is valuable and results from reflecting the free non-protein-bound pharmacologically active component in the serum [13, 32].

One example is valproic acid, used not only in the treatment of epilepsy but also in psychiatry. It is used in schizophrenia along with other medications and as a second-line treatment for bipolar disorder. Drug determination in saliva can be a simple test checking whether the patient is taking the drugs systematically as well as drug toxicity. It also makes it possible to determine the approximate level in the serum without blood sampling [33]. Dwivedi et al. [34] showed that the mean ratio of saliva to serum-free valproic acid concentration indicates that the saliva levels can predict the free drug concentrations in serum, and it also shows the protein binding of valproic acid in both. Carbamazepine, methadone, nicotine, cocaine, amphetamines, or buprenorphine has also been measured in oral fluid [13, 32, 35].

## 3. Dementia

Recent studies showed that saliva might be a valuable marker of neurodegenerative diseases [36–39].

An example is dementia, which is characterized by progressive cognitive impairment and behavioral changes. There are five types of dementia, for now, namely, Alzheimer's disease, vascular dementia, Lewy body dementia, frontotemporal dementia, and mixed dementias [36, 38]. It is estimated that about 50% of all dementia instances are Alzheimer's disease [36, 39], in which amyloid *β* and tau protein accumulate in the central nervous system.

Amyloid *β* is one of the most significant sources of reactive oxygen species in patients with dementia. It is deposited in the brain and also in the peripheral regions like the nasal mucosa, lacrimal glands, or lingual glands (salivary gland epithelium cells) [24, 36].

It is proved that oligomer forms of amyloid *β* activate nicotinamide adenine dinucleotide phosphate-oxidase (NADPH), increase the formation of hydrogen peroxide, and increase reactive oxygen species production in the mitochondria. This happens through modulation of alcohol dehydrogenase activity, which binds *α*-ketoglutarate dehydrogenase and amyloid *β*. Accumulation of amyloid *β* in the secretory epithelium of salivary glands in patients with dementia disrupts the local redox balance and is responsible for damage to the structure and function of salivary glands [24, 36]. Changes in the composition of saliva can involve worsening in the quality of life of patients with dementia. These changes may cause problems with swallowing, inflammatory and fungal lesions, and worse cavital digestion [24, 36, 40, 41].

It is possible that oxidative stress is a significant factor that might cause dysfunction of the salivary glands. Scientists compare this to the mechanism observed in metabolic syndromes, such as insulin resistance [36, 42], obesity [36, 43], and diabetes [36, 44, 45], or autoimmune diseases, such as Sjögren syndrome and rheumatoid arthritis [36, 46]. The newest studies show that saliva might be an alternative diagnostic material to blood plasma or serum. In cases of dementia, it is used as an indicator of redox homeostasis biomarkers [24, 36, 40]. Choromańska et al. [36] proved decreased antioxidant properties of saliva and increased levels of DNA products in dementia patients. Moreover, they showed oxidative damage of protein and lipid, with simultaneously reduced secretion of nonstimulated and stimulated saliva. They suggested that changes in salivary redox homeostasis are independent of systemic changes in the progression of dementia [36].

## 4. Alcohol Dependence

Alcohol consumption is a serious public health problem and has been associated with high mortality rates. The world's population of adults suffering from alcohol abuse is estimated at about 4.9%. More than 2% of the world's population is alcohol dependent, while in Europe, it is estimated at 4% and in America 3.4% [47]. The World Health Organization assessed that the problem of binge drinking concerns more than 7% of the world's population (over 16% in Europe and 13% in America). In the last years, binge drinking has become the dominant pattern of alcohol consumption among adults [47].

So far, some chronic alcohol markers have been found in saliva, namely, aminotransferases and gamma-glutamyl-transferase, ethanol, sialic acid, hexosaminidase A, and glucuronidase. Waszkiewicz et al. [11, 47, 48] suggested that alcohol such as methanol, diethylene, ethylene, and glycol and salivary glycoproteins like oral peroxidase, *α*-amylase, clusterin, haptoglobin, heavy and light chains of immunoglobulins, and transferrin may be possible alcohol markers. In addition, chronic drinking leads to disturbances in adaptive and innate immunities, like immunoglobulin A, peroxidase, and lactoferrin [11, 48].

Waszkiewicz et al. [1, 49] found increased activity or concentration of *β*-hexosaminidase and immunoglobulin A in binge drinking [1, 49]. They also showed specific changes in salivary immunity in binge drinkers and alcohol-dependent patients. Furthermore, it was showed that even a single high dose of alcohol (2 g/kg) increases the level of salivary immunoglobulin A [2, 50]. Binge drinking caused disturbances in innate salivary immunity (lysozyme). They found possible applicability of raised immunoglobulin A concentration and oral peroxidase activity in binge and chronic drinking differentiation [2, 50].

## 5. Autism Spectrum Disorders

Autism spectrum disorder is a neurological and developmental disorder that affects communication and behavior [51]. It is included in the group of developmental disorders because symptoms begin early in childhood, mostly appearing in the first three years of life [52]. Scientists estimate the prevalence of autism spectrum disorders as 6 per 1,000. However, the frequency rates vary for each of the developmental disorders in the spectrum [52]. Early diagnosis and intervention might improve functional outcomes in children with autism spectrum disorder. Diagnosis, prognosis, and monitoring of symptoms of autism spectrum disorder can also be helped with biomarkers [53].

Ngounou Wetie et al. [53] tried to optimize salivary proteomic biomarker methods and to identify initial biomarkers in children with autism spectrum disorders. They assumed that mass spectrometry-based proteomics could help expose biomarkers for autism spectrum disorder. Scientists have analyzed the salivary proteome in individuals with autism spectrum disorders compared to control subjects. They found statistically significant differences in several salivary proteins, e.g., the elevation of prolactin-inducible protein, lactotransferrin, Ig kappa chain C region, Ig gamma-1 chain C region, Ig lambda-2 chain C regions, neutrophil elastase, and polymeric immunoglobulin receptor and deletion in malignant brain tumors 1. Their achievement supports the concept that immune system and gastrointestinal disturbances may be present in individuals with autism spectrum disorders [53].

Bhandary and Hari [54] studied the role of saliva as a biomarker and oral health status of children with autism spectrum disorders. They observed that salivary pH and buffering capacity were lower in children with autism spectrum disorders than their healthy siblings [54].

In another study, the authors measured salivary microRNA. They assumed that epigenetic mechanisms including microRNAs might contribute to the autism spectrum disorder phenotype by changing the neurodevelopmental gene networks. They showed the presence of the differential expression of 14 microRNAs (e.g., miR-628-5p, miR-27a), which are expressed in the developing brain. Furthermore, the impact of microRNAs on brain development and its correlates with neurodevelopmental behaviors were shown. MicroRNAs found in saliva showed high specificity and cross-validated utility. MicroRNAs seem to be a potential screening tool for autism spectrum disorders [55].

## 6. Neuroendocrine System

The use of saliva for monitoring steroid hormone levels has received increasing attention in recent years. The monitoring of steroid hormone levels is currently commercially available. There is nothing unusual in that, since levels of salivary steroid hormones reflect the free and thus the active level of these hormones in the blood [56]. The levels of cortisol, dehydroepiandrosterone, estradiol, estriol, progesterone, testosterone, etc. can be accurately assessed in saliva, being useful in evaluations of mood and cognitive-emotional behavior, in the diagnosis of premenstrual depression, to assess ovarian function, to evaluate risk for preterm labor and delivery, in full-term and preterm neonate monitoring, to study child health and development, as well as to predict sexual activity in adolescent males, or in Cushing's syndrome screening.

Protein hormones are too large to reach saliva through passive diffusion and can reach saliva through contamination from serum as a result of the outflow of gingival crevicular fluid or from oral wounds [14]. Protein hormones are therefore not useful in routine salivary analyses. Archunan et al. [57] presented that cyclic variations in salivary levels of glycosaminoglycans (GAGs) and sialic acid (SA) as well as in steroid (estrogens, progesterone) and glycoprotein (luteinizing hormone, LH) hormones can be helpful in predicting ovulation. SA and GAG content showed a distinct peak at ovulation during a normal menstrual cycle. Such hormonal changes in estrogen levels and a peak in LH might be the reason for proteoglycan degradation. Estrogen can inhibit the synthesis of the extracellular matrix, shifting normal proteoglycan turnover toward degradation processes. Identification of the period of ovulation in humans is critical in the treatment of infertility, which may result in mental disorders [21, 57, 58]. An easy, new, and noninvasive method of ovulation detection may help in the infertility treatment. Besides the salivary hormonal changes, changes in salivary GAGs and SA seem to show promise in the identification of the period of ovulation as well as the assessment of endocrine function.

Cortisol plays an important role as a marker of psychiatric disorders, such as anxiety and depression. Changes in cortisol levels appear in response to stress as well as emotional support. Chronic stress may lead to disease by activating the hypothalamic-pituitary-adrenocortical (HPA) axis. The correlation of cortisol levels in blood and saliva is extremely strong, and the noninvasive quantification of this hormone in saliva meets the detection criteria in biomedical research, both scientific and diagnostic [59–61].

Another parameter that is very helpful in assessing a neurotic disorder is alpha-amylase, which reflects catecholamines in the blood. Therefore, it reflects stress levels, reacting even faster than cortisol [62, 63].

Thus, further studies focusing on changes in salivary components during different physiological and pathophysiological states seem to be warranted.

## 7. Conclusions

Based on these properties, human saliva has successfully been used in the diagnosis of many systemic diseases, like cancers (ovarian, lung, breast, and pancreatic), autoimmune diseases (Sjögren's syndrome, celiac disease, and Hashimoto's thyroiditis), infectious diseases (HIV, hepatitis, and malaria), and endocrinological diseases (types 1 and 2 diabetes, Cushing's syndrome) as well as diseases of the gastrointestinal tract (gastroesophageal reflux disease). Also, it is used in toxicological diagnostics, drug monitoring, and forensic medicine. The usefulness of saliva as a biological marker has also been extended to psychiatry. Saliva is recommended as an excellent material for biochemical, toxicological, and immunological diagnostics of not only oral cavity or systemic diseases but also in the still unexplored field of neuropsychiatry.
